# Recovery of information from multiple imputation: a simulation study

**DOI:** 10.1186/1742-7622-9-3

**Published:** 2012-06-13

**Authors:** Katherine J Lee, John B Carlin

**Affiliations:** 1 Clinical Epidemiology and Biostatistics Unit, Murdoch Childrens Research Institute, The Royal Children’s Hospital, Flemington Road, Parkville, VIC, 3052, Australia; 2 Department of Paediatrics, The University of Melbourne, Melbourne, VIC, 3010, Australia

**Keywords:** Missing data, Multiple imputation, Fully conditional specification, Multivariate normal imputation, Non-normal data

## Abstract

**Background:**

Multiple imputation is becoming increasingly popular for handling missing data. However, it is often implemented without adequate consideration of whether it offers any advantage over complete case analysis for the research question of interest, or whether potential gains may be offset by bias from a poorly fitting imputation model, particularly as the amount of missing data increases.

**Methods:**

Simulated datasets (n = 1000) drawn from a synthetic population were used to explore information recovery from multiple imputation in estimating the coefficient of a binary exposure variable when various proportions of data (10-90%) were set missing at random in a highly-skewed continuous covariate or in the binary exposure. Imputation was performed using multivariate normal imputation (MVNI), with a simple or zero-skewness log transformation to manage non-normality. Bias, precision, mean-squared error and coverage for a set of regression parameter estimates were compared between multiple imputation and complete case analyses.

**Results:**

For missingness in the continuous covariate, multiple imputation produced less bias and greater precision for the effect of the binary exposure variable, compared with complete case analysis, with larger gains in precision with more missing data. However, even with only moderate missingness, large bias and substantial under-coverage were apparent in estimating the continuous covariate’s effect when skewness was not adequately addressed. For missingness in the binary covariate, all estimates had negligible bias but gains in precision from multiple imputation were minimal, particularly for the coefficient of the binary exposure.

**Conclusions:**

Although multiple imputation can be useful if covariates required for confounding adjustment are missing, benefits are likely to be minimal when data are missing in the exposure variable of interest. Furthermore, when there are large amounts of missingness, multiple imputation can become unreliable and introduce bias not present in a complete case analysis if the imputation model is not appropriate. Epidemiologists dealing with missing data should keep in mind the potential limitations as well as the potential benefits of multiple imputation. Further work is needed to provide clearer guidelines on effective application of this method.

## Introduction

Statistical analysis of epidemiological data is often hindered by missing data. Multiple imputation is a two-stage process whereby missing values are imputed multiple times from a statistical model based on the available data and used in analyses that combine results across the multiply imputed datasets [1,2]. Such an approach is used increasingly to deal with missing data [3,4], but it is often carried out without carefully considering the extent of likely gain over a complete case analysis nor whether there is the potential to introduce bias from a poorly fitting imputation model.

A common misconception with multiple imputation arises from focussing on the mechanics of filling in missing values, as if imputation recovers a fully observed sample, when in fact the value of multiple imputation (if any) relates to whether it recovers information about (population) parameters of interest. Information recovery may be in the form of reduced bias or increased precision, and a challenge for the analyst is to assess whether using multiple imputation for a given problem presents sufficient potential for recovery of information to be worthwhile.

Once it has been concluded that multiple imputation may be of value, the question becomes whether the available multiple imputation technology will provide a valid approach. There are currently two readily available methods for generating imputed datasets. Multivariate normal imputation (MVNI) uses a Markov Chain Monte Carlo algorithm to obtain imputed values assuming a multivariate normal distribution for all variables subject to missingness [2]. An alternative is fully conditional specification (FCS) where separate regression models are fitted for each variable with missingness, conditional on other variables in the imputation model [5,6]. Both approaches assume values are “missing at random” (MAR), i.e. the missingness is dependent on observed values only, and rely on parametric assumptions, in particular that continuous variables are normally distributed (at least conditionally under FCS). If data truly are MAR then multiple imputation using an appropriate imputation model is a valid approach (asymptotically unbiased with correct standard errors and coverage [1]), so the accuracy of results is determined by the validity of the assumptions made in the imputation model [7]. We note that this paper leaves aside the important issue of potential sensitivity of results to the MAR assumption itself [8,9].

Both MVNI and FCS assume specific parametric models that do not fit real data perfectly. Our recent paper demonstrated the inadequacy of both of these multiple imputation approaches in estimating regression coefficients in a standard regression analysis when there was missingness in a highly skewed covariate when the non-normality was not taken into account in the imputation model [10]. The failure of model assumptions is likely to be more damaging as the amount of missingness increases, since more data will be generated from an ill-fitting model [7].

The aim of this paper is to explore the recovery of information from multiple imputation, and how this is affected by the fraction of observations with missing values. We report the results of a simulation study in which we generate missingness assuming various forms of MAR, so that multiple imputation would be valid if performed under a correct model, and compare inferences for regression parameters under various missing data scenarios between multiple imputation and complete case analysis. In our previous paper we demonstrated that MVNI and FCS produced similar results [10] and hence for this analysis we focus on MVNI. In interpreting the results from the simulation study we focus on what gains can be made by using multiple imputation compared to complete case analysis for estimating the effect of a binary exposure, and how the potential gains are affected by which variables contain missing values. As a secondary aim, we also explore how potential benefits of imputation are affected by the fit of the imputation model, extending the results of our previous paper [10] to explore the effect of ignoring skewness while imputing in the presence of increasing fractions of missing data.

### Analysis

#### Creating the simulated datasets

As in previous work [10], we use data from a synthetic “population” of 971,327 girls created to resemble a real epidemiological study [11]. The analysis included six variables representing data collected at the time of recruitment (Wave I): race (black, non-black Hispanic and other), school grade (ordinal: years 7 [aged 12–13] to 11 [aged 16–17]), self reported health (ordinal: 1 = excellent, 2 = very good, 3 = good, 4 = fair, 5 = poor) and fitness (ordinal – you are physically fit: 1 = strongly agree, . . ., 5 = strongly disagree), emotional distress (continuous 0–3 with higher scores representing higher distress), and the primary exposure of interest, a binary indicator for whether the girl had dieted in the previous 7 days or not. The outcome was the emotional distress score measured at a second wave of follow-up (Wave II) one year later (again continuous 0–3). Datasets were created by drawing random samples of 1000 observations from the synthetic population (with replacement between samples) and we focus on the estimation of a regression model for emotional distress at Wave II:

(1) $$\begin{array}{l} E\left(ldistW2\right)=\alpha +\beta_{1}diet+\beta_{2}ldistW1\\ \;+\beta_{3}race_{1}+\beta_{4}race_{2}+\beta_{5}grade\\ \;+\beta_{6}health+\beta_{7}fitness \end{array}$$

where *diet* represents the dieting indicator, *race*_*1*_ and *race*_*2*_ are indicators for being black and non-black Hispanic respectively, and *grade*, *health* and *fitness* represent the three categorical variables. Emotional distress (at Waves I and II) was highly positively skewed and therefore was analysed on the log_e_ scale (denoted *ldistW1* and *ldistW2* respectively). As in previous work, we artificially inflated the diet effect so that it was borderline statistically significant with the chosen sample size [10].

In a second set of analyses we categorised the outcome distress at Wave II into depressed and not depressed (by dichotomising at an arbitrary threshold of 1), and focussed on estimating the parameters of the multiple logistic regression:

(2) $$\begin{array}{l} \text{distW2i}∼\text{binomial}\left(1,p\right)\\ \text{logit}\left(p\right)=\varphi +\eta_{1}\text{diet}+\eta_{2}\text{ldistW}1+\eta_{3}{\text{race}}_{1}\\ \;+\eta_{4}{\text{race}}_{2}+\eta_{5}\text{grade}+\eta_{6}\text{health}+\eta_{7}\text{fitness} \end{array}$$

where *distW2i* is the indicator for being distressed at Wave II (=1 if *distW2* > 1 and 0 otherwise) and other variables are as previously described.

### Inducing missingness

We considered the simple situation where data were MAR in a single variable in two scenarios, where there was induced missingness in (i) the baseline distress measure, a strong predictor of outcome, and (ii) the dieting indicator, the exposure of interest.

In both settings, values were set to missing with a probability determined by a logistic regression model. Missingness in distress at Wave I was determined by:

(3) $$\begin{array}{l} \text{logit Pr}\left(distW1\;\text{missing}\right)\\ \;=\gamma +\delta_{1}diet+\delta_{2}race_{1}+\delta_{3}race_{2}+\delta_{4}grade\\ \;+\delta_{5}ldistW2+0\times ldistW1 \end{array}$$

and in diet by:

(4) $$\begin{array}{rl} \text{logit Pr}\;\left(diet\;\text{missing}\right)= & \varphi +0\times diet\\ \;+\delta_{2}race_{1}+\delta_{3}race_{2}\\ \;+\delta_{4}grade+\delta_{5}ldistW2\\ \;+\delta_{6}ldistW1 \end{array}$$

In each case *δ*_*2*_ = log(2.7), *δ*_*3*_ = log(2.7), *δ*_*4*_ = log(1.2), and *δ*_*5*_ = log(1.3), with *δ*_*1*_ = log(2.7) in (3) and *δ*_*6*_ = log(1.3) in (4). *γ* and *φ* were adjusted to obtain 10%, 25%, 50%, 75% and 90% missingness in Equations (3) and (4) respectively.

In the analyses of the binary depression outcome, missingness in distress at Wave I was induced using the model:

(5) $$\begin{array}{l} \text{logit Pr}\left(distW1\text{missing}\right)\\ \;=\varphi +\delta_{1}diet+\delta_{2}race_{1}+\delta_{3}race_{2}+\delta_{4}grade\\ \;+\delta_{7}distW2i \end{array}$$

where *δ*_*1*_, *δ*_*2*_, *δ*_*3*_, *δ*_*4*_ were as above, *δ*_*7*_ = log(2.7) and φ was adjusted to control the amount of missingness.

### Analysis of the simulated data

For each simulated dataset, with missingness imposed according to Equation (3) or (4), we estimated the regression model of interest (Equation 1) using MVNI and a complete case analysis, and similarly for the second set of analyses with missingness imposed according to Equation (5) and the analysis model given in Equation (2). All analyses were performed in Stata version 11 [12], with MVNI carried out using “mi impute mvn” with a uniform prior distribution.

MVNI was performed including all covariates from the analysis model (Equation 1) and the outcome in the imputation model, to ensure the maximum recovery of information. Distress at Waves I and II, and the health and fitness scores were transformed to improve normality using either a simple log transformation or a shifted log transformation (“log-skew0”) *u = ln(±x - k)*, choosing *k* and the sign of *x* so that *u* has zero skewness as used previously [10]. Imputed values of distress from the log-skew0 transformation were truncated at the low end at the smallest observed value in the sample, with high values from both methods truncated at the scale maximum of 3. Grade was included as a linear predictor in the imputation model.

### Comparison of methods

The properties of the regression coefficient estimates from each analysis were assessed by comparing the results from the 1000 simulated datasets (each of 1000 observations) to the “true” population parameters from the synthetic population of 971,327. In each multiple imputation analysis, 20 imputed datasets were created, with inferences for the coefficients obtained by combining results over the imputed datasets using Rubin’s rules [1].

We report the bias, the average (estimated) standard error (SE), the standardised bias (calculated as the bias divided by the average SE), the mean squared error (MSE), and the coverage of the estimated 95% confidence interval (CI) compared to the population parameters, as described previously [10]. Based on the simulation sample size of 1000, the Monte Carlo (MC) error of the estimates across the repeated samples can be calculated as $\text{SE}/\sqrt{1000}$, and the estimated coverage should lie in the range 93.6% to 96.4% (with 95% probability), if the true coverage is equal to the nominal 95%. There is also MC error due to the finite number of imputations, but this additional uncertainty was small compared with the magnitude of the estimates [13]. Findings were similar for each of the health and fitness covariates, so only the results for the health effect are reported.

### Results from the simulation study

Table 1 displays pairwise Spearman rank correlations between the continuous and ordinal variables in the analysis model for the complete synthetic population, to provide a simple description of bivariate associations between these variables (notwithstanding the limitations of correlations for categorical variables). In the population, girls who dieted tended to be more distressed at Wave I, be in a slightly higher grade, and have higher self-reported health and fitness compared to non-dieters, although these relationships were fairly weak. There were slightly larger correlations between health and fitness and distress at Wave I (correlations both 0.22). In relation to outcome, there were similar levels of distress at Wave II in dieters and non-dieters (correlation = −0.0008) and a moderate correlation between distress at Wave I and outcome (correlation = 0.56). For a fuller description of this dataset see [10]. 

**Table 1:** Spearman rank correlations between covariates and distress at Wave II in the synthetic population (n = 971,327)

	**Diet**	**Log (distress at Wave I)**	**Grade**	**Health**	**Fitness**
Log(Distress WI)	0.07				
Grade	0.06	0.10			
Health	0.07	0.22	0.03		
Fitness	0.13	0.22	0.09	0.42	
**Outcomes**					
Log(Distress WII)	−0.0008	0.56	0.07	0.20	0.20

### Scenario 1: Continuous outcome – missing data on distress at wave I

When missing data were introduced in distress at Wave I (Table 2) and 90% of data were complete, there was slight bias in the exposure of interest, the dieting coefficient, in the complete case analysis. This bias was eliminated using multiple imputation irrespective of the transformation used to address the non-normality. There was little bias in the estimates for the other parameters for all analyses. For all parameters, precision was improved using multiple imputation, although gains in precision were small with this low rate of missingness (SE for the diet coefficient 8% larger with complete case analysis).

**Table 2:** Performance of methods for regression of (continuous) distress at Wave II, with missing baseline distress

**% complete data**	**Method**	**Diet (*β*_1_ = −0.101)**				**Distress (*β*_2_ = 0.554)**				**Health (*β*_6_ = 0.042)**			
		**Bias**	**SE**	**StdBias**	**Coverage**	**Bias**	**SE**	**StdBias**	**Coverage**	**Bias**	**SE**	**StdBias**	**Coverage**
90%	CCA	−0.016	0.069	−0.229	93.9%	<0.001	0.036	0.014	95.1%	0.002	0.032	0.063	93.7%
	MVNI-log	−0.001	0.064	−0.012	93.0%	−0.009	0.035	−0.252	95.1%	0.003	0.031	0.095	94.1%
	MVNI-skew0	−0.001	0.064	−0.010	93.2%	<0.001	0.035	0.001	95.4%	0.002	0.031	0.061	94.2%
75%	CCA	−0.031	0.082	−0.383	92.4%	−0.009	0.040	−0.219	95.4%	0.001	0.036	0.016	95.3%
	MVNI-log	0.004	0.067	0.059	95.1%	−0.032	0.037	−0.863	84.7%	0.003	0.032	0.110	94.7%
	MVNI-skew0	0.003	0.066	0.047	94.5%	−0.009	0.038	−0.233	96.0%	0.001	0.031	0.033	94.5%
50%	CCA	−0.054	0.118	−0.458	91.9%	−0.011	0.051	−0.210	95.5%	0.003	0.048	0.064	94.9%
	MVNI-log	0.002	0.075	0.031	94.6%	−0.064	0.045	−1.426	68.7%	0.008	0.034	0.246	94.5%
	MVNI-skew0	<0.001	0.074	−0.003	95.2%	−0.012	0.046	−0.263	94.7%	0.002	0.033	0.050	94.8%
25%	CCA	−0.058	0.190	−0.308	91.8%	−0.020	0.075	−0.265	93.7%	0.002	0.071	0.031	94.8%
	MVNI-log	0.004	0.091	0.041	95.4%	−0.106	0.060	−1.775	57.4%	0.013	0.039	0.328	93.9%
	MVNI-skew0	0.004	0.092	0.045	94.6%	−0.023	0.059	−0.397	90.7%	0.003	0.039	0.072	94.4%
10%	CCA	−0.023	0.345	−0.068	94.8%	−0.025	0.125	−0.196	95.9%	0.008	0.120	0.071	96.0%
	MVNI-log	0.011	0.129	0.084	96.0%	−0.145	0.092	−1.576	60.9%	0.021	0.049	0.423	91.3%
	MVNI-skew0	0.013	0.136	0.096	95.1%	−0.056	0.091	−0.614	89.5%	0.009	0.052	0.183	94.8%

Measures of performance are mean values from the estimation of the *β* parameters in Equation 1 across the 1000 simulated datasets of 1000 observations (compared to the true values from the synthetic population of 971,327). CCA = Complete Case Analysis; MVNI = Multivariate normal imputation; StdBias = standardised bias; SE = average (estimated) standard error across the 1000 datasets.

**Figure 1:**
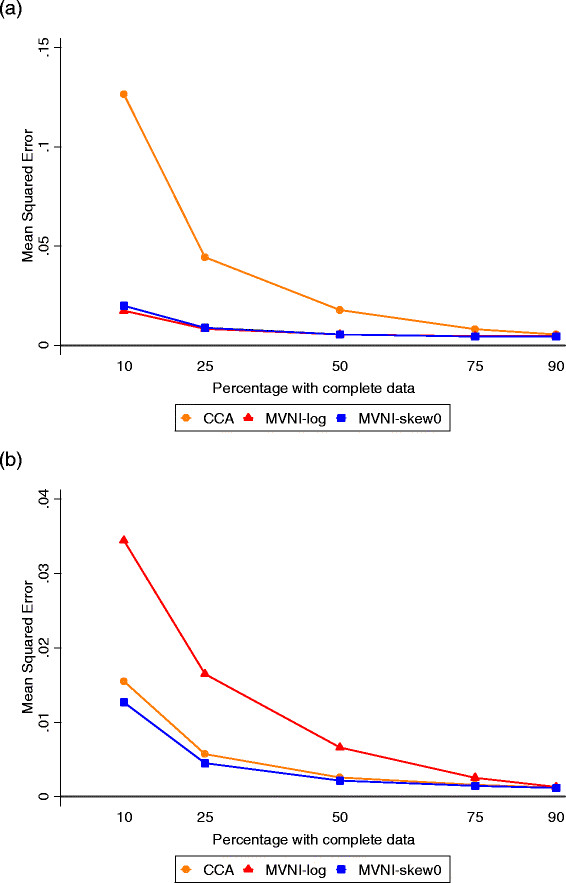
**Mean Squared Errors from regression of (continuous) distress at Wave II with missing baseline distress. a**) Diet, **b**) Emotional Distress at Wave I Results presented are the average Mean Squared Error across the 1000 simulated datasets in the parameter estimates from linear regression of (continuous) emotional distress at Wave II from Equation 1, with missing data on emotional distress at baseline. CCA = Complete Case Analysis; MVNI = Multivariate normal imputation.

When 75% of data were observed, both imputation models led to smaller bias and improved precision in the dieting coefficient compared with complete case analysis (SE approximately 24% larger) corresponding to a smaller MSE (Figure 1), although even complete case analysis had a reasonably small bias (standardised bias = −0.031/0.082 = −0.38) [14]. There were also gains in estimation of the health coefficient, a variable with some correlation with distress at Wave I, although these gains were less pronounced than in the dieting coefficient, which was only weakly correlated with distress at Wave I. In contrast, there was larger bias in the coefficient for distress at Wave I (which contained missing data) when a (simple) log transformation was used in the imputation model, compared with complete case analysis (standardised bias = −0.86, compared to −0.22), problems which were ameliorated when the log-skew0 transformation was used for imputation. It is difficult to interpret SEs in the presence of bias, but for the least biased method, using the log-skew0 transformation, there were much smaller gains in precision from multiple imputation in the distress coefficient (SE approximately 5% larger with complete case analysis) than with the other parameters. A similar pattern was observed when 50% of data were complete.

When only 25% of data were complete, there was slightly less bias and substantially greater precision in the dieting coefficient under multiple imputation compared with complete case analysis (SE approximately 2 times larger with complete case analysis), reflected in a much smaller MSE (Figure 1). In contrast, there was much larger bias (bias = −0.106, MC error = 0.002) and considerable under-coverage of the 95% CIs for the distress coefficient from multiple imputation using a log transformation compared with complete case analysis. Although the bias from multiple imputation was improved using the log-skew0 transformation, the coverage from this analysis was still below the nominal 95% with this large amount of missing data. There was also large bias (0.013, MC error = 0.001) and under-coverage of CIs for the health coefficient, a covariate correlated with the variable subject to missingness, when the simple log transformation was used in the imputation model. However, the bias and coverage were similar for the health coefficient from multiple imputation and complete case analysis when the log-skew0 transformation was used, with 82% gain in precision and a lower MSE under multiple imputation.

A similar pattern was observed when only 10% of data were complete, with even larger gains in bias and precision in the dieting coefficient (e.g. SE 2.5 times larger with complete case analysis compared with the MVNI-skew0 method), but large bias and reduced coverage for the distress coefficient from both imputation analyses compared to complete case analysis. Again this affected the estimation of the health coefficient under multiple imputation using the log-transformation but not using the log-skew0 transformation, where there was similar bias to the complete case analysis but a 2.3-fold reduction in the SE.

### Scenario 2: Continuous outcome – missing data on diet

**Figure 2:**
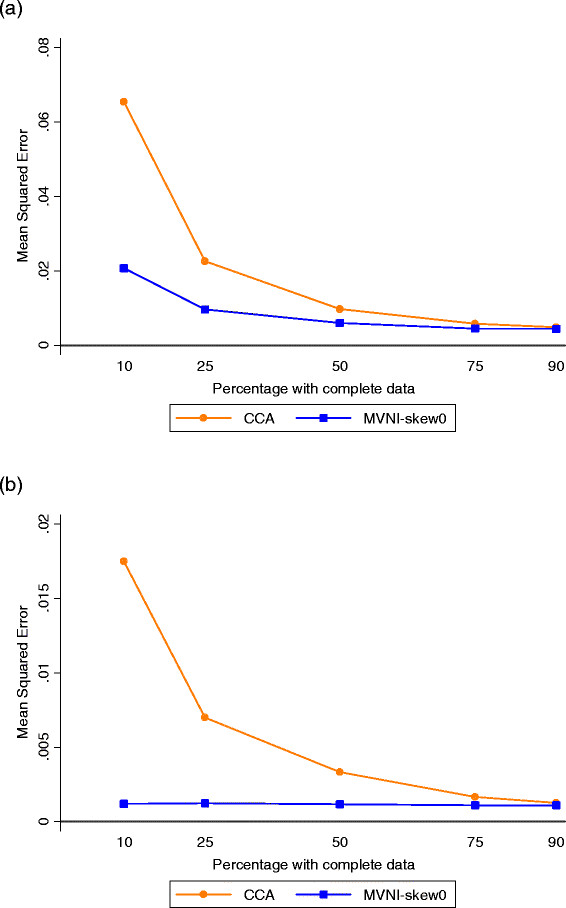
**Mean Squared Errors from regression of (continuous) distress at Wave II with missing dieting indicator. a**) Diet, **b**) Emotional Distress at Wave I Results presented are the average Mean Squared Error across the 1000 simulated datasets in the parameter estimates from linear regression of (continuous) emotional distress at Wave II from Equation 1, with missing data on the dieting indicator. CCA = Complete Case Analysis; MVNI = Multivariate normal imputation.

When missingness was imposed in the exposure of interest, the dieting indicator, bias and coverage for estimation of its effect were fairly similar under complete case and multiple imputation irrespective of the amount of missing data (Table 3, Figure 2; note that only multiple imputation results using the log-skew0 transformation are presented because of its superior performance in the previous scenario). There were relatively modest gains in precision and MSE from multiple imputation compared with complete case analysis for the dieting coefficient even when a large proportion of observations were incomplete (SE 68% larger with complete case analysis when only 10% of data were available). Reduced bias, improved coverage and a smaller MSE were obtained under multiple imputation for all coefficients aside from diet, with larger gains in precision as the amount of missing data increased, for example gains of over 3-fold were obtained for all coefficients (except diet) compared to complete case analysis when only 10% of data were available.

**Table 3:** Performance of methods for regression of (continuous) distress at Wave II with missing dieting indicator

**% complete data**	**Method**	**Diet (*β*_1_ = −0.101)**				**Distress (*β*_2_ = 0.554)**				**Health (*β*_6_ = 0.042)**			
		**Bias**	**SE**	**StdBias**	**Coverage**	**Bias**	**SE**	**StdBias**	**Coverage**	**Bias**	**SE**	**StdBias**	**Coverage**
90%	CCA	0.001	0.067	0.019	94.2%	−0.006	0.036	−0.154	94.9%	−0.001	0.032	−0.020	95.1%
	MVNI-skew0	0.005	0.065	0.074	94.6%	0.001	0.034	0.020	94.7%	−0.001	0.030	−0.043	95.3%
75%	CCA	−0.001	0.075	−0.014	95.0%	−0.014	0.040	−0.360	94.9%	<0.001	0.036	0.009	95.5%
	MVNI-skew0	0.008	0.070	0.119	96.1%	<0.001	0.034	0.013	95.2%	−0.001	0.030	−0.042	95.6%
50%	CCA	−0.006	0.097	−0.065	94.2%	−0.028	0.049	−0.577	91.8%	0.004	0.047	0.086	94.6%
	MVNI-skew0	0.014	0.080	0.175	95.6%	0.001	0.034	0.032	94.6%	<0.001	0.030	−0.002	96.3%
25%	CCA	−0.011	0.147	−0.076	94.6%	−0.043	0.072	−0.603	91.9%	−0.001	0.071	−0.014	94.3%
	MVNI-skew0	0.018	0.104	0.172	96.1%	<0.001	0.034	−0.009	93.6%	−0.002	0.030	−0.071	94.2%
10%	CCA	−0.020	0.250	−0.081	95.8%	−0.053	0.118	−0.452	92.2%	0.009	0.120	0.077	94.7%
	MVNI-skew0	0.011	0.149	0.071	95.0%	−0.001	0.035	−0.042	95.9%	<0.001	0.031	−0.014	95.4%

Measures of performance are mean values in the estimation of the *β* parameters from Equation 1 across the 1000 simulated datasets of 1000 observations (compared to the true values from the synthetic population of 971,327). CCA = Complete Case Analysis; MVNI = Multivariate normal imputation; StdBias = standardised bias; SE = average (estimated) standard error across the 1000 datasets.

### Scenario 3: Binary outcome – missing data on distress at wave I

With the dichotomised outcome and missingness in distress at Wave I, there was reduced bias in the dieting coefficient, and improved precision and a reduced MSE for the diet and health coefficients under multiple imputation, compared to complete case analysis, when at least 50% of data were fully observed (Table 4). However, there was large bias (−0.043, MC error = 0.007) in the distress coefficient from multiple imputation even when as much as 75% of data were observed. When only 25% of data were observed, both complete case analyses and multiple imputation produced gross under-coverage of the 95% CI for all parameters, despite a similar pattern of reduced bias in the diet coefficient and smaller SEs in the diet and health coefficients under multiple imputation compared with complete case analysis. It was not possible to obtain results with only 10% of data complete due to a large number of datasets with zero counts in the cross-tabulation of diet and distress at Wave II.

**Table 4:** Performance of methods for regression of (dichotomous) distress at Wave II with missing baseline distress

**% with complete data**	**Method**	**Diet (η_1_ = 0.754)**				**Distress (η_2_ = 6.260)**				**Health (η_6_ = 1.132)**			
		**Bias**	**SE**	**StdBias**	**Coverage**	**Bias**	**SE**	**StdBias**	**Coverage**	**Bias**	**SE**	**StdBias**	**Coverage**
90%	CCA	−0.115	0.257	−0.449	92.6%	0.021	0.197	0.106	95.0%	0.007	0.115	0.065	95.0%
	MVNI-skew0	0.002	0.226	0.008	93.9%	−0.003	0.199	−0.013	95.3%	0.005	0.106	0.048	95.1%
75%	CCA	−0.209	0.319	−0.653	91.9%	0.013	0.227	0.059	93.70%	0.006	0.133	0.047	95.4%
	MVNI-skew0	−0.007	0.233	−0.030	95.8%	−0.043	0.230	−0.187	94.6%	0.004	0.108	0.039	94.3%
50%	CCA	−0.243	0.485	−0.501	96.3%	0.046	0.306	0.149	94.5%	0.008	0.180	0.043	94.0%
	MVNI-skew0	0.014	0.251	0.057	95.9%	−0.082	0.304	−0.270	94.6%	0.008	0.115	0.066	95.6%
25%	CCA	−0.120	0.833	−0.144	82.4%	0.091	0.487	0.188	79.4%	0.021	0.286	0.073	80.0%
	MVNI-skew0	0.021	0.308	0.068	80.6%	−0.155	0.447	−0.348	78.5%	0.002	0.132	0.012	80.5%

Measures of performance are mean values in the estimation of the *β* parameters from Equation 1 across the 1000 simulated datasets of 1000 observations (compared to the true values from the synthetic population of 971,327). CCA = Complete Case Analysis; MVNI = Multivariate normal imputation; StdBias = standardised bias; SE = average (estimated) standard error across the 1000 datasets.

Note in this example it was not possible to include an analysis where only 10% of the data were complete due to a large number of datasets with zero counts in the cross-tabulation of diet and distress at Wave II.

## Summary of results and discussion

In this study we explored the value of multiple imputation in a simulated regression analysis and examined how this varied according to the pattern of missing data. We found that although multiple imputation recovered important information about associations concerning fully observed variables when there were large amounts of missing data in a covariate, much smaller gains in bias and precision were seen in the coefficient for the variable with missing values, irrespective of whether the outcome was continuous or binary. The important difference is that when there is missing data in confounding variables only, we lose potentially available information about the relationship between the fully observed variables (including the variable of interest) and the outcome, which can be recovered by imputation. In contrast, cases for which the variable of interest is missing hold little information about the relationship between that variable and the outcome, so little information is gained on the coefficient for the variable of interest when we impute missing values.

Similar findings of variable benefits of multiple imputation were observed by White and Carlin [15] who suggested that when associations are weak, the gain in precision for a regression coefficient, calculated as:

(6) $${\left(\frac{SE_{\text{CC}}}{SE_{\text{MI}}}\right)}^{2}-1$$

where *SE*_*CC*_ and *SE*_*MI*_ are the SEs from the complete case and multiple imputation analysis respectively, might be approximated by the fraction of incomplete cases (observations) among those with observed values of the independent variable of interest (“FICO”). This concept is consistent with our finding that gains in information arise primarily when cases with observed values for the exposure of interest are recovered by imputing missing values in other covariates. In our dataset we had strong predictors of missingness and we saw large gains in precision compared to the FICO, which perhaps provides a lower bound for potential gains.

Potential information recovery from multiple imputation needs to be weighed up against the possibility of bias being introduced by a poorly fitting imputation model. A second aim of this study was to extend our previous work which focussed on detailed comparisons between the MVNI and FCS imputation methods [10], to explore the extent to which the performance of multiple imputation was affected by a poorly fitting model as the amount of missing data increased. We found that although multiple imputation was fairly robust to non-normality when there were few missing values, there was large bias and poor coverage in the estimation of the regression coefficient for the highly skewed continuous covariate when the variable was subject to substantial missingness, with similar results for the continuous and binary outcome settings. In both settings, there was also some contamination in the estimation of coefficients of other variables, particularly those that were correlated with the variable subject to missingness, if the skewness was not removed. Relating back to our previous paper [10], it may be worth noting that we obtained an essentially identical pattern of results for all of the scenarios examined when multiple imputation was carried out using the FCS method (fitted using the ice command in Stata again using a log and log-skew0 transformation to address non-normality).

The results presented in this paper extend our previous findings that bias can be introduced if skewness is not adequately addressed, highlighting the increasing unreliability of multiple imputation methods if skewness is not removed as the proportion of incomplete observations increases and a larger fraction of data is imputed from a mis-specified model [7]. In particular, when the majority of observations had missing data, multiple imputation under the normal model became unreliable even if the skewness was removed. A similar finding of larger bias when there was a larger proportion of missing data was seen by Demirtas et al. [16] who explored the performance of MVNI in a simulation study with incomplete data in various skewed and multimodal variables. However, in contrast to our findings, their overall conclusion was that MVNI performed reasonably well even when the normality assumption was clearly violated, particularly when there was a large sample size.

It has been shown that multiple imputation has greater value if variables used in the imputation model are predictive of missingness and of the missing values [14]. However, the results from the current study suggest that multiple imputation can introduce bias in the coefficients for variables that are correlated with the covariate with missing data when skewness was not removed during imputation, particularly when there are lots of missing values, as seen with the health and fitness coefficients in the first example. There was less of an impact in estimating the dieting effect since the diet indicator was not associated with distress at Wave I.

Researchers often ask how much missing data can be imputed. Marshall et al. [17] reported multiple imputation to be useful when up to 50% of observations had data MAR in one or more variables in their simulations. Barzi et al. [18] found inflated variability and convergence problems with multiple imputation when more than 60% of observations had missing data, with varying results, depending on the method of multiple imputation, when 10-60% of observations had missing data. As we have demonstrated here, whether multiple imputation may offer substantial benefit over complete case analysis depends on whether missing data is in the exposure of interest or in covariates, and also on the strength of inter-relationships between variables. These factors, along with the predictors of missingness and the missing data pattern, vary considerably across settings making it impossible to specify general rules for when multiple imputation will be beneficial (aside from the dangers of an ill-specified model). The relative merits of multiple imputation and complete case analysis were discussed by White and Carlin [15] who showed that when data were missing in just one or two covariates neither multiple imputation nor complete case was universally better than the other, depending on the missing data mechanism, although they did find multiple imputation was superior in a wider range of the settings examined. While it does not seem possible to set rules for when multiple imputation will be beneficial it is important to identify situations where multiple imputation is unlikely to be helpful.

### Limitations of this simulation study

Caution is needed in generalising from the results of a single simulation study. In particular these results are affected by the inherent structure of the synthetic population used. However, this example clearly illustrates potential dangers of using multiple imputation when there are large amounts of missingness and/or highly skewed data. We have considered the simple situation of data MAR in a single variable, but in practice there is likely to be missingness in a number of variables, with complex inter-relationships between variables and their missingness patterns, which may have a range of consequences. Finally the analysis presented in this paper missingness was highly dependent on a few (known) variables, relationships which are likely to be weaker in practice, so reducing the potential for information recovery. Further exploration in more complex scenarios, for example with more variables subject to missingness and different patterns of missing data, would be beneficial.

## Conclusions

The results from this study demonstrate that although it may be important to use multiple imputation to recover information when there are missing data in covariates required for adjustment, multiple imputation has substantially less value when there are missing data (even when MAR) in the exposure of interest. This study also highlights the potential for poor results from standard approaches to multiple imputation particularly when a large fraction of individuals have missing data.

These findings have important implications, particularly for large epidemiological studies where there may be varying degrees of missingness across a number of variables. Firstly, these results demonstrate that when there is a lot of missing data, large-scale imputation may be futile, and in particular it can introduce more bias than a complete case analysis if the imputation model does not fit the data well. For this reason when using multiple imputation we would recommend carrying out a complete case analysis in parallel, as suggested by White and Carlin [15] – this provides reassurance if inference from the two are similar, but may highlight issues with one or both approaches if results differ substantially. Secondly these results suggest that it is important to develop tailored imputation models to address specific analysis questions. This recommendation clashes with the idea that multiple imputation could be carried out by an “expert imputer” to create a set of imputed datasets that could be used by data analysts to answer a range of research questions [1,19].

Our overall conclusion is that the current wave of enthusiasm for multiple imputation should be tempered with greater caution about its limitations. Further work is needed to elucidate more detailed guidelines for its effective application.

## Competing interests

The authors declare that they have no competing interests.

## Authors’ contributions

KJL planned and carried out the analysis in the paper and took a lead in writing the manuscript with substantial input from JBC throughout. Both authors read and approved the final manuscript.

## References

[B1] Rubin DB Multiple imputation for nonresponse in surveys 1987 New York: Wiley

[B2] Schafer JL Analysis of Incomplete Multivariate Data 1997 London: Chapman & Hall

[B3] Klebanoff MA Cole SR Use of Multiple Imputation in the Epidemiologic Literature Am J Epidemiol 2008 168 355 357 10.1093/aje/kwn071 18591202 PMC2561989

[B4] Sterne JAC White IR Carlin JB Royston P Kenward MG Wood AM Carpenter JR Multiple imputation for missing data in epidemiological and clinical research: potential and pitfalls BMJ 2009 338 b2393 10.1136/bmj.b2393 19564179 PMC2714692

[B5] Raghunathan TE Lepkowski JM Van Hoewyk J Solenberger P A multivariate technique for multiply imputing missing values using a sequence of regression models Survey Methodology 2001 27 85 95

[B6] VanBuuren S Boshuizen HC Knook DL Multiple imputation of missing blood pressure covariates in survival analysis Statistics in Medicine 1999 18 681 694 10.1002/(SICI)1097-0258(19990330)18:6<681::AID-SIM71>3.0.CO;2-R 10204197

[B7] Rubin D Multiple imputation after 18+ years J Am Stat Assoc 1996 91 473 489 10.1080/01621459.1996.10476908

[B8] Carpenter JR Kenward MG Missing data in clinical trials – a practical guide 2008 Birmingham: National Health Service Coordinating Centre for Research Methodology Available from http://www.haps.bham.ac.uk/publichealth/methodology/docs/invitations/Final_Report_RM04_JH17_mk.pdf

[B9] Carpenter JR Kenward MG White IR Sensitivity analysis after multiple imputation under missing at random: a weighting approach Statistical Methods in Medical Research 2007 16 259 275 10.1177/0962280206075303 17621471

[B10] Lee KJ Carlin JB Multiple imputation for missing data: fully conditional spacification versus multivariate normal imputation Am J Epidemiol 2010 171 624 632 10.1093/aje/kwp425 20106935

[B11] Schafer JL Kang JDY Average causal effects from nonrandomized studies: A practical guide and simulated example Psychological Methods 2008 13 279 313 19071996 10.1037/a0014268

[B12] StataCorp Stata: Release 11. Statistical Software 2009 College Station, TX: StataCorp LP

[B13] Royston P Carlin JB White IR Multiple imputation of missing values: new features for “mim” Stata J 2009 9 252 264

[B14] Collins LM Schafer JL Kam C A comparison of inclusive and restrictive strategies in modern missing data procedures Psychological Methods 2001 6 330 351 11778676

[B15] White I Carlin J Bias and efficiency of multiple imputation compared with complete-case analysis for missing covariate data Statistics in Medicine 2010 29 2920 2931 10.1002/sim.3944 20842622

[B16] Demirtas H Freels SA Yucel RM Plausibility of multivariate normality assumption when multiply imputing non-Gaussian continuous outcomes: a simulation assessment J Stat Comput Simul 2008 78 69 84 10.1080/10629360600903866

[B17] Marshall A Altman DG Royston P Roger LH Comparison of techniques for handling missing covariate data within prognostic modelling studies: a simulation study BMC Medical Research Methodology 2010 10 7 10.1186/1471-2288-10-7 20085642 PMC2824146

[B18] Barzi F Woodward M Imputation of missing values in practice: results from imputations of serum cholesterol in 28 cohort studies Am J Epidemiol 2004 160 34 45 10.1093/aje/kwh175 15229115

[B19] Rubin DB Schenker N Multiple imputation in health-care databases: an overview and some applications Statistics in Medicine 1991 10 585 598 10.1002/sim.4780100410 2057657

